# Supplementary material for: Early conversion to a CNI-free immunosuppression with SRL after renal transplantation—Long-term follow-up of a multicenter trial
Source: PLoS One. 2020 Aug 5;15(8):e0234396. doi: 10.1371/journal.pone.0234396 (PMC7406080; doi:10.1371/journal.pone.0234396)
Supplement: S1 Protocol — (DOC) [file pone.0234396.s011.doc]

**Investigator Initiated Research**

**Grant synopsis**

**“Longterm follow-up analysis of patients included in the SMART-trial with special focus on the development of Donor-specific-Antibodies**

**(DSA)”**

**Investigator-team:** **Prof. Dr. Joachim Andrassy**

Dr. Teresa Kauke

Prof. Dr. Marcus Guba

**Contact Information:**Chirurgie- Allgemein/HPB/ & Transplantationschirurgie

Ludwig-Maximilians-Universität

Klinikum Grosshadern

Marchioninistr. 15

81377 München

Germany

Phone: +49-089/7095-0

Mobile +49-089/7095-0 #1233

Email: [Joachim.Andrassy@med.uni-muenchen.de](mailto:Joachim.Andrassy@med.uni-muenchen.de)

**Summary:**

Early replacement of Calcineurin-inhibitors (CNI) by mTOR-inhibitors results in improved renal function 12 and 36 months post renal transplantation (data shown for Sirolimus in our SMART-study). Development of donor-specific HLA antibodies (DSA) has been identified

as risk factor for chronic rejection and graft failure for patients under standard Immunosuppression. A recent monocentric analysis conducted in patients on a second

mTOR-inhibitor Everolimus (EVRL), has demonstrated increased levels of donor-specific HLA antibodies compared with a CsA-based regimen (1). In contrast, in the CONCEPT-trial, another early initiation study using Sirolimus, less HLA antibodies have been found in the

SRL group compared to CsA (2), a result with limited significance due to patient selection and missing information whether antibodies were de novo DSA. No further analyses have been conducted so far. Here, we aim to conduct a long term follow-up of patients of our

SMART trial, with special focus on donor specific HLA antibodies. To our knowledge this is the first analysis investigating systematically the long-term development of DSA using a multicenter approach.

**Background:**

The SMART trial investigated the effect of an early CNI replacement (7-21 days post transplant) with the mTOR-inhibitor Sirolimus after renal transplantation. Pts. were either switched to SRL + MMF + ST or remained on CsA + MMF + ST. Immediatly after the CNI withdrawal transplant function significantly improved and remained improved throughout the study duration. The development of donor-specific HLA antibodies (DSA) post transplantation has been associated with higher chronic graft failure rates (1). Antibody mediated rejections following DSA development have also been described (1, 2). Moreover, the risk of graft dysfunction and graft loss is significantly increased when cellular rejection coincides with DSA and antibody mediated microvascular injury (3). Data with respect to DSA development under SRL vs. CNIs remain highly inconsistent. Some postulate that SRL is a risk factor for DSA development (4), whereas others report lower DSA levels under the mTOR-inhibitor (5). In a monocenter investigation of patients from two EVRL trials (ZEUS,

HERAKLES) seven out of 65 (10.8%) patients on cyclosporine developed DSA after a median of 991 days. In comparison, 14 out of 61 patients (23.0%) randomized to EVRL developed DSA after 551 days (log-rank: p = 0.048). There was also a higher rate of antibody mediated rejection within the EVRL group (8 vs. 2 Patients (log-rank: p = 0.036). In a long-term follow-up of the CONCEPT trial at 4 and 5 years there is a trend

towards reduced HLA-antibody development in SRL treated patients. For this project we have the outstanding opportunity to receive DSA data from a randomized set of patients (SMART-Study). Furthermore, apart from the DSA all other relevant data (safety, tolerability and immunosuppressive effect) will be retrieved which means long term data for SRL in

renal transplantation (around 6 years post transplantation).

**Preliminary results:**

We investigated in a single center approach the documentation of DSA development within our SMART patient records. Herein we found a trend towards less DSA in SRL treated patients. The incidence was 1/10 SRL patients vs. 6/24 CsA patients, that developed DSA over different time point within their clinical follow-up.

**Study type:**

Investigational study, with in part retrospective safety analysis

**Study design:**

It is the aim to include the majority of randomized patients within the SMART trial in to the analysis. All patients will be invited to participate in this investigation at a routine visit, for control of kidney function, health parameters and a full laboratory profile.

Patient records will be investigated toward former side effects, acute rejection and change in immunosuppression.

**Sample size and participating centers:**

In the SMART core study 140 patients were included, the 36m follow up covered 123 patients. Carrying forward these numbers, a realistic estimate of patients participating in this analysis is about 100.

SMART Study group centers:

1 Department of Surgery, Munich University Hospital, Campus Grosshadern, Munich, Germany.

2 Department of General, Visceral, and Transplantation Surgery, Charite, Campus Virchow-Clinic, Department of Medicine, Division of Medicine, Charite University, Berlin, Germany

3 Department of Medicine, Division of Nephrology, University of Erlangen, Erlangen, Germany.

4 Department of Internal Medicine II, Nephrology and Transplantation, University Medical Center, Regensburg, Germany.

5 Department of Urology, University of Rostock, Rostock, Germany.

6 Department of Surgery, University of Münster, Münster, Germany.

**Primary and secondary endpoint:**

**Primary**

1. determination of DSA status & class characterization at time point of examination regardless of follow-up time since transplantation (ITT).

**Secondary**

1. determination of DSA status & class characterization at time point of examination regardless of follow-up time since transplantation (o.T.).
2. timepoint of first detection of DSA after Tx , if available
3. correlation of DSA development with underlying Immunosuppression (uni/multivariate) (ITT/o.T)
4. correlation of DSA development with previous acute rejection
5. multivariate analysis of other risk factors
6. kidney function (ITT/o.T)
7. safety profile (ITT/o.T)

**Methods:**

During routine clinical visits of the patients, not necessarily at one of the primary study centers, kidney function and health parameters will be assessed and a full laboratory profile will be run. As for transplant recipients there is a medical need for a closely monitored outpatient setting, these examinations are expected to be completed within a short period over at least 2 months. The study is classified as interventional because the analysis of DSA status requiring an additional blood sample is not covered by clinical routine.

All serum samples will be qualitatively screened for HLA antibodies by the Luminex-based bead assay LABScreen Mixed (One Lambda, Canoga Park, CA, USA). Donor specificity of HLA antibodies will be determined by LABScreen Single Antigen beads (One Lambda). All tests will be performed according to the manufacturer’s guidelines in a central HLA lab of the investigators’ institution.

Characterization of HLA-Antibodies will include:

Class I: HLA-A, -B, -Cw

Class II: HLA-DRB1, -DQB, -DQA, -DPB, -DPA

Definition of donor specitivity will be done by comparison with DONOR HLA typing and classification as de novo if panel-reactivity = 0 before Tx.

Patient records will be screened for safety parameters esp. SAEs since the last visit of the SMART core study (12 months).

**Statistical Analyses:**

All analyses were by intention to treat (ITT). Comparisons between treatment groups were performed with the chi-squared test for categorical data, Wilcoxon–Mann–Whitney test

for continuous data, and cumulative incidence plots with a log-rank test for time-to-event data. A p-value <0.05 was considered to be statistically significant.

An on treatment (o.T.) analysis will be performed separately for all relevant parameters, especially for DSA development, kidney function and malignancies.

**Administration:**

An independent institutional review board (IRB) approval will be requested. Due to interventional character the study must be approved by German regulatory body BfArM.

Informed consent will be obtained for all patients.

**Trial duration:**

The investigation will be conducted within 6 months, including full data analysis.

**Publication:**

Planned publication in a peer reviewed journal (Transplantation/TI etc) short after data collection/analysis – Q4 2014.

**Significance:**

This could be the first detailed analysis of DSA development within a RCT with an early initiation of SRL towards a CNI-free based maintenance Immunosuppression. This is of scientific and clinical meaningful value, as DSA development has been identified impacting graft survival. This might be also of relevance in current discussions of findings of elevated DSA development in a EVRL based regimen and the immunosuppressive value of different mTOR-inhibitors.

**References:**

1. Ekberg H, Tedesco-Silva H, Demirbas A, et al. Reduced exposure to calcineurin inhibitors in renal transplantation. N Engl J Med 2007; 357: 2562.
2. Guba M, Pratschke J, Hugo C, et al. Renal function, efficacy, and safety of sirolimus and mycophenolate mofetil after short-term calcineurin inhibitor-based quadruple therapy in de novo renal transplant patients: one-year analysis of a randomized multicenter trial. Transplantation 2010; 90: 175.
3. Guba M, Pratschke J, Hugo C et al.; SMART-Study Group. Early conversion to a sirolimus-based, calcineurin-inhibitor-free immunosuppression in the SMART trial: observational results at 24 and 36 months after transplantation. Transpl Int. 2012 Apr;25(4):416-23.
4. Lebranchu Y, Thierry A, Toupance O, et al. Efficacy on renal function of early conversion from cyclosporine to sirolimus 3 months after renal transplantation: concept study. Am J Transplant 2009; 9: 1115.
5. Lebranchu Y, Thierry A, Thervet E et al.; Effi cacy and safety of early cyclosporine conversion to sirolimus with continued MMF-four-year results of the Postconcept study. Am J Transplant. 2011 Aug; 11(8): 1665-1675.
6. Budde K, Becker T, Arns W, et al. Everolimus-based, calcineurin-inhibitor-free regimen in recipients of de-novo kidney transplants: an open-label, randomised, controlled trial. Lancet 2011; 377: 837.
7. Schena FP, Pascoe MD, Alberu J, et al. Conversion from calcineurin inhibitors to sirolimus maintenance therapy in renal allograft recipients: 24-month efficacy and safety results from the CONVERT trial. Transplantation 2009; 87: 233.
8. Liefeldt L, Brakemeier S, Glander P, et al.. Donor-specific HLA antibodies in a cohort comparing everolimus with cyclosporine after kidney transplantation. Am J Transplant. 2012 May;12(5):1192-8.
9. Pascual J, Arns W. Does everolimus increase donor-specific HLA antibodies in kidney transplant recipients? Am J Transplant. 2012 Sep;12(9):2561-2; author reply 2563.
10. Loupy A, Hill GS, Jordan SC. The impact of donor-specific anti-HLA antibodies on late kidney allograft failure. Nat Rev Nephrol. 2012 Apr 17;8(6):348-57.
11. Wiebe C, Gibson IW, Blydt-Hansen TD, et al. Evolution and clinical pathologic correlations of de novo donor-specific HLA antibody post kidney transplant. Am J Transplant. 2012 May;12(5):1157-67.
12. Cantarovich D, De Amicis S, Akl A, Devys A, Vistoli F, Karam G, Soulillou JP.Posttransplant donor-specific anti-HLA antibodies negatively impact pancreas transplantation outcome. Am J Transplant. 2011 Dec;11(12):2737-46.
13. Sánchez-Fructuoso AI, Santiago JL, Pérez-Flores I, et al. De novo anti-HLA antibodies in renal allograft recipients: a cross-section study. Transplant Proc. 2010 Oct;42(8):2874-6.
14. Tait BD, Süsal C, Gebel HM, et al. Consensus guidelines on the testing and clinical management issues associated with HLA and non-HLA antibodies in transplantation. Transplantation. 2013 Jan 15;95(1):19-47.
