# Supplementary material for: Early conversion to a CNI-free immunosuppression with SRL after renal transplantation—Long-term follow-up of a multicenter trial
Source: PLoS One. 2020 Aug 5;15(8):e0234396. doi: 10.1371/journal.pone.0234396 (PMC7406080; doi:10.1371/journal.pone.0234396)
Supplement: S1 Table — (DOCX) [file pone.0234396.s012.docx]

**S1 Table: Therapy discontinuations and changes to Tacrolimus**

A significantly greater percentage of SRL patients were switched to TAC vs CsA. Exposure time to Tacrolimus was not significantly different between the groups.

|  | A: SRL (N=38) | B: CsA (N=33) | P-value |
| --- | --- | --- | --- |
| Therapy discontinuation n (%) | 26 (68.4) | 11 (33.3) | 0.004 |
| Change to TAC n (%) | 20 (52.6) | 8 (24.2) | 0.017 |
| TAC therapy duration (mo) | 72.3+31.8 | 51.7+25.8 | 0.09 |
