# Supplementary material for: Early conversion to a CNI-free immunosuppression with SRL after renal transplantation—Long-term follow-up of a multicenter trial
Source: PLoS One. 2020 Aug 5;15(8):e0234396. doi: 10.1371/journal.pone.0234396 (PMC7406080; doi:10.1371/journal.pone.0234396)
Supplement: S2 Table — (DOCX) [file pone.0234396.s013.docx]

**S2 Table:** Reasons for therapy changes to Tacrolimus

| Category | | | |  | A: SRL | B: CsA | |
| --- | --- | --- | --- | --- | --- | --- | --- |
| Rejection | | | |  | 2 | 2 | |
| Deteriorating Tx function | | | |  | 8 | 4 | |
| Rising Creatinine | | | |  | 4 | 1 | |
| Fibrosis, progressive degeneration | | | |  | 1 | 0 | |
| Proteinuria | | | |  | 1 | 0 | |
| Recurrence underlying disease | | | |  | 1 | 2 | |
| unclear | | | |  | 1 | 1 | |
| Miscellaneous | | | |  | 10 | 2 | |
| Diarrhea | | | |  | 0 | 2 | |
| Hyperlipidemia | | | |  | 1 | 0 | |
| HLA-antibodies | | | |  | 2 | 0 | |
| Wound complications | | | |  | 2 | 0 | |
| Pain lower legs | | | |  | 2 | 0 | |
| Pneumonitis | | | |  | 2 | 0 | |
| Leuco-/Thrombocytopenia | | | |  | 1 | 0 | |
|  |  |  |  | | | |  |
|  |  |  |  | | | |  |
|  |  |  |  | | | |  |
|  |  |  |  | | | |  |
|  |  |  |  | | | |  |
|  |  |  |  | | | |  |
|  |  |  |  | | | |  |
|  |  |  |  | | | |  |
|  |  |  |  | | | |  |
|  |  |  |  | | | |  |
|  |  |  |  | | | |  |
|  |  |  |  | | | |  |
|  |  |  |  | | | |  |
|  |  |  |  | | | |  |
|  |  |  |  | | | |  |
|  |  |  |  | | | |  |
|  |  |  |  | | | |  |
|  |  |  |  | | | |  |
|  |  |  |  | | | |  |
|  |  |  |  | | | |  |
|  |  |  |  | | | |  |
|  |  |  |  | | | |  |
|  |  |  |  | | | |  |
|  |  |  |  | | | |  |
|  |  |  |  | | | |  |
|  |  |  |  | | | |  |
|  |  |  |  | | | |  |
|  |  |  |  | | | |  |
|  |  |  |  | | | |  |
|  |  |  |  | | | |  |
