# Supplementary material for: Early conversion to a CNI-free immunosuppression with SRL after renal transplantation—Long-term follow-up of a multicenter trial
Source: PLoS One. 2020 Aug 5;15(8):e0234396. doi: 10.1371/journal.pone.0234396 (PMC7406080; doi:10.1371/journal.pone.0234396)
Supplement: S3 Table — A. Mixed model analysis of eGFR (Nankivell). B. Mixed model analysis of eGFR (MDRD). (DOCX) [file pone.0234396.s014.docx]

**S3a Table:** Mixed Model Analysis of eGFR (Nankivell).

| Solution for Fixed Effects | | | | | | | |
| --- | --- | --- | --- | --- | --- | --- | --- |
| Effect | Estimate | Standard Error | Lower | Upper | DF | t Value | Pr > \|t\| |
| Intercept | 59.8466 | 3.6944 | 52.4766 | 67.2167 | 69 | 16.20 | <.0001 |
| Treatment (SRL) | 4.6989 | 5.0568 | -5.3892 | 14.7870 | 69 | 0.93 | 0.3560 |
| Time (M) | -0.06176 | 0.02476 | -0.1105 | -0.01306 | 348 | -2.49 | 0.0131 |
| Time (M)*Treatment (SRL) | 0.06646 | 0.03361 | 0.000354 | 0.1326 | 348 | 1.98 | 0.0488 |

SRL treatment over time significantly impacts transplant function as measured by Nankivell.

**S3b Table:** Mixed Model Analysis of eGFR (MDRD).

| Solution for Fixed Effects | | | | | | | |
| --- | --- | --- | --- | --- | --- | --- | --- |
| Effect | Estimate | Standard Error | Lower | Upper | DF | t Value | Pr > \|t\| |
| Intercept | 53.8866 | 3.2583 | 47.3865 | 60.3868 | 69 | 16.54 | <.0001 |
| Treatment (SRL) | 1.2484 | 4.4575 | -7.6441 | 10.1409 | 69 | 0.28 | 0.7803 |
| Time (M) | -0.07927 | 0.02546 | -0.1293 | -0.02921 | 381 | -3.11 | 0.0020 |
| Time(M)*Treatment (SRL) | 0.06340 | 0.03468 | -0.00479 | 0.1316 | 381 | 1.83 | 0.0683 |

SRL treatment over time impacts transplant function as measured by MDRD.
