# Supplementary material for: Early conversion to a CNI-free immunosuppression with SRL after renal transplantation—Long-term follow-up of a multicenter trial
Source: PLoS One. 2020 Aug 5;15(8):e0234396. doi: 10.1371/journal.pone.0234396 (PMC7406080; doi:10.1371/journal.pone.0234396)
Supplement: S4 Table — (DOCX) [file pone.0234396.s015.docx]

**S4 Table:** Cox model for patient and death censored graft survival

|  | Analysis of Maximum Likelihood Estimates | | | | | | | | |
| --- | --- | --- | --- | --- | --- | --- | --- | --- | --- |
|  | Parameter | DF | Parameter Estimate | Standard Error | Chi-Square | Pr > ChiSq | Hazard Ratio | 95% Hazard Ratio Confidence Limits | |
| DCGS | ARM=SRL | 1 | -0.77433 | 0.42905 | 3.2571 | 0.0711 | 0.461 | 0.199 | 1.069 |
| Pat. survival | ARM=SRL |  | 0.20279 | 0.47447 | 0.1827 | 0.6691 | 1.225 | 0.483 | 3.104 |

*According to this analysis the hazard ratio for graft loss was 0.461 (95%CI: 0.199-1.069) in patients once randomized to SRL compared to the CsA group.* There is no difference regarding the patient survival.
