# Supplementary material for: Early conversion to a CNI-free immunosuppression with SRL after renal transplantation—Long-term follow-up of a multicenter trial
Source: PLoS One. 2020 Aug 5;15(8):e0234396. doi: 10.1371/journal.pone.0234396 (PMC7406080; doi:10.1371/journal.pone.0234396)
Supplement: Attachment — Submitted filename: Andrassy SMART Response.docx [file pone.0234396.s016.docx]

| Klinikum der Universität München ∙ Klinik für Allgemein-, Viszeral-, Transplantations-chirurgie ∙ CAMPUS GROSSHADERN ∙ Marchioninistr. 15 ∙ D-81377 München | |  | **Prof. Dr. med. J. Andrassy**  [Joachim.andrassy@med.uni-muenchen.de](mailto:Joachim.andrassy@med.uni-muenchen.de)  Sekretariat  Tel.: +49 89 4400-76561  Fax: +49 89 4400-78894  <http://gch.klinikum.uni-muenchen.de>  Postanschrift:  Klinikum der Universität München  Klinik für Allgemein-, Viszeral-, Transplantationschirurgie  Marchioninistr. 15  D-81377 München |
| --- | --- | --- | --- |
| Prof. Justyna Gołębiewska  Academic Editor  Plos ONE | |  |  |
| Ihr Zeichen: | Unser Zeichen: |  | München, den 07.03.20 |

PONE-D-19-25265
Early conversion to a CNI-free immunosuppression with SRL after renal Transplantation – long-term follow-up of a multicenter trial
PLOS ONE

Dear Prof. Dr. Andrassy,

Thank you for submitting your manuscript to PLOS ONE. After careful consideration, we feel that it has merit but does not fully meet PLOS ONE’s publication criteria as it currently stands. Therefore, we invite you to submit a revised version of the manuscript that addresses the points raised during the review process.

==============================
ACADEMIC EDITOR:

The manuscript is of potential interest to the renal transplant community. However, it is not acceptable for publication in its current form. The major limitation of the study is a substantial drop-out rate and considerable tacrolimus exposure in both groups. Therefore additional data should be provided and discussion expanded:

- The Authors should evaluate and compare the mean time with tacrolimus exposure in SRL vs CsA arm. Why were so many patients switched to tacrolimus?  A small table listing the reasons would be informative. Just listing late adverse events is not as informative as listing those that caused a change in drug delivery.

*We included data to the patients that were switched to Tacrolimus (see supplementary Tables).*

- Data on proteinuria in both groups should be provided.

*We included information on proteinuria (see Table 7)*

- The Authors should highlight the limitations of the very low rate of patients on the original immunosuppression at the end of 8-9 years follow-up.

*This has been addressed in the discussion*

- The conclusions about dnDSA must consider that the majority of the patients were in use of tacrolimus that was not the original immunosuppression.

*This has been addressed in the discussion*

- The absence of histology of the grafts also limits the ability to make firm conclusions on the impact of CNI free status on graft stability, this limitation should be provided as well.

*This has been addressed in the discussion*

Additionally, please provide corrections according to the statistical Reviewer suggestions (Reviewer 6).

==============================

We would appreciate receiving your revised manuscript by Jan 23 2020 11:59PM. When you are ready to submit your revision, log on to <https://www.editorialmanager.com/pone/> and select the 'Submissions Needing Revision' folder to locate your manuscript file.

If you would like to make changes to your financial disclosure, please include your updated statement in your cover letter.

To enhance the reproducibility of your results, we recommend that if applicable you deposit your laboratory protocols in protocols.io, where a protocol can be assigned its own identifier (DOI) such that it can be cited independently in the future. For instructions see: <http://journals.plos.org/plosone/s/submission-guidelines#loc-laboratory-protocols>

Please include the following items when submitting your revised manuscript:

- A rebuttal letter that responds to each point raised by the academic editor and reviewer(s). This letter should be uploaded as separate file and labeled 'Response to Reviewers'.
- A marked-up copy of your manuscript that highlights changes made to the original version. This file should be uploaded as separate file and labeled 'Revised Manuscript with Track Changes'.
- An unmarked version of your revised paper without tracked changes. This file should be uploaded as separate file and labeled 'Manuscript'.

Please note while forming your response, if your article is accepted, you may have the opportunity to make the peer review history publicly available. The record will include editor decision letters (with reviews) and your responses to reviewer comments. If eligible, we will contact you to opt in or out.

We look forward to receiving your revised manuscript.

Kind regards,

Justyna Gołębiewska
Academic Editor
PLOS ONE

Journal Requirements:

**When submitting your revision, we need you to address these additional requirements:**

1. **Please ensure that your manuscript meets PLOS ONE's style requirements, including those for file naming. The PLOS ONE style templates can be found at** <http://www.plosone.org/attachments/PLOSOne_formatting_sample_main_body.pdf> **and** <http://www.plosone.org/attachments/PLOSOne_formatting_sample_title_authors_affiliations.pdf>
2. Please provide additional details regarding participant consent. In the ethics statement in the Methods and online submission information, please ensure that you have specified (1) whether consent was suitably informed and (2) what type you obtained (for instance, written or verbal). If your study included minors under age 18, state whether you obtained consent from parents or guardians. If the need for consent was waived by the ethics committee, please include this information.

*Information has been added in the Methods section accordingly.*

3. In your Methods section, please provide additional information regarding the donated tissues or organs used in the study. Please specify whether the study involved the use of donated tissue/organs from any vulnerable populations, and provide information on the consent given by the donor or their next of kin. Examples of vulnerable populations include prisoners, subjects with reduced mental capacity due to illness or age, and children. If such a population as used, please ensure you have describe the population and justify the decision to use tissue/organ donations from this group. If not, please state in your Ethics Statement, 'None of the transplant donors were from a vulnerable population and all donors or next of kin provided written informed consent that was freely given.'

*Information has been added in the Methods section accordingly*

4. Thank you for including your ethics statement:  "This trial was conducted according to GCP guidelines and the declaration of Helsinki and was approved by the local ethics committees of the participating centers."

a.Please amend your current ethics statement to include the full name of the ethics committee/institutional review board(s) that approved your specific study.

*Information has been added in the Methods section accordingly*

b.Once you have amended this/these statement(s) in the Methods section of the manuscript, please add the same text to the “Ethics Statement” field of the submission form (via “Edit Submission”).

*Information has been added accordingly*

For additional information about PLOS ONE ethical requirements for human subjects research, please refer to <http://journals.plos.org/plosone/s/submission-guidelines#loc-human-subjects-research>.

5. Please amend the manuscript submission data (via Edit Submission) to include author Andreas Pascher.

6.Thank you for stating the following in the Financial Disclosure section:

J. A. received funding from an unrestricted medical grant by Pfizer Pharma GmbH to perform this trial.

The funders had no role in study design, data collection and analysis, decision to publish, or preparation of the manuscript.

We note that you received funding from a commercial source: Pfizer

Please provide an amended Competing Interests Statement that explicitly states this commercial funder, along with any other [relevant declarations](http://www.plosone.org/static/competing.action) relating to employment, consultancy, patents, products in development, marketed products, etc.

Within this Competing Interests Statement, please confirm that this does not alter your adherence to all PLOS ONE policies on sharing data and materials by including the following statement: "This does not alter our adherence to PLOS ONE policies on sharing data and materials.” (as detailed online in our guide for authors <http://journals.plos.org/plosone/s/competing-interests>).  If there are restrictions on sharing of data and/or materials, please state these. Please note that we cannot proceed with consideration of your article until this information has been declared.

Please include your amended Competing Interests Statement within your cover letter. We will change the online submission form on your behalf.

7.

We note that you have indicated that data from this study are available upon request. PLOS only allows data to be available upon request if there are legal or ethical restrictions on sharing data publicly. For information on unacceptable data access restrictions, please see <http://journals.plos.org/plosone/s/data-availability#loc-unacceptable-data-access-restrictions>.

In your revised cover letter, please address the following prompts:

a) If there are ethical or legal restrictions on sharing a de-identified data set, please explain them in detail (e.g., data contain potentially identifying or sensitive patient information) and who has imposed them (e.g., an ethics committee). Please also provide contact information for a data access committee, ethics committee, or other institutional body to which data requests may be sent.

b) If there are no restrictions, please upload the minimal anonymized data set necessary to replicate your study findings as either Supporting Information files or to a stable, public repository and provide us with the relevant URLs, DOIs, or accession numbers. Please see <http://www.bmj.com/content/340/bmj.c181.long> for guidelines on how to de-identify and prepare clinical data for publication. For a list of acceptable repositories, please see <http://journals.plos.org/plosone/s/data-availability#loc-recommended-repositories>.

We will update your Data Availability statement on your behalf to reflect the information you provide.

Review Comments to the Author

Please use the space provided to explain your answers to the questions above. You may also include additional comments for the author, including concerns about dual publication, research ethics, or publication ethics. (Please upload your review as an attachment if it exceeds 20,000 characters)

**Reviewer #1**: This is long-term data about the SMART study. This study aims to evaluate the eGFR in patients early converted to CNI-free immunosuppression with SRL after renal Transplantation. The authors discussed that were few studies with long term outcome about CNI-free immunosuppression.

This is a well writing manuscript with an extended follow-up of the primary study. The extension aims to evaluate the development of de novo donor-specific antibodies (dnDSA). This is a relevant question because the immunosuppression without CNI was associated with higher rates of dnDSA. A serious limitation was the sample size with very low patients in use of the original immunosuppression (31.6% in the sirolimus and 66.7% in the ciclosporin)

Main Questions:

1. Possible the good results of early convesion to sirolimus was related to the induction therapy with thymoglobulin. This type of induction therapy diverges from other CNI-convertions trials. The authors should highlight this point. Additionally, the use of a loading dose of sirolimus is not common practice today.

*This trial was designed in 2005/2006. The reviewer is right that loading doses of SRL are no longer common practice nowadays. Induction therapy was* ***not*** *Thymoglobulin but ATG-Fresenius (nowadays Grafalon, Neovii). As has been described in the primary 12 month’s report we’ve had a considerable percentage of acute rejections in the first patients. Therefore, an amendment was introduced thereby increasing the dose of ATG-F from 4 to 9mg/kg BW.*

*ATG-F may not be as potent as Thymoglobulin as indicated by a few trials (Styrc B in Clin Transpl 2019 or Opelz G in Transplantation 2006).*

*Altogether the existing evidence is not subtle enough to discuss possible advantages or disadvantages of ATG-F over the other induction therapies.*

2. Measures of dnDSA. The authors show that there were no differences in dnDSA between the ciclosporin and the sirolimus group. However, this could be analysed with caution because only 31.6% of patients in the sirolimus group were in use of the original immunosuppression. The majority of the patients were switched to tacrolimus. The authors should evaluate the mean time with tacrolimus exposure.

*This is a limitation of this study. We have included information on the patients switched to Tacrolimus in the supplementary material* *(see supplementary Tables 1 and 2) and we discussed this limitation in more detail.*

3. Transplant Function. The low sample size does not allow to show better renal function in the sirolimus group. Only the Nankivell formula in the ITT and on the therapy population showed the difference. I suggest the conclusion based only the more contemporaneous formula as MDRD. Addictionally, the analysis should be done by **mixed models**. This type of analysis consider the repeated measures of the data.

*As the SMART-Trial was designed in 2005/2006, the Nankivell Formula was used as primary parameter and it had been used in the two publications to the SMART-Trial before. Therefore, for comparability and continuity we feel the necessity to show Nankivell data here, as well. Nonetheless, all other more contemporaneous formulas as MDRD and CKD-EPI have been included in the Tables 5 and 6.*

The best method is as longitudinal data analysis of covariance with na interaction between time and the treatment variable
Ref: Different ways to estimate treatment effects in randomised controlled trials. Contemp Clin Trials Commun. 2018 Mar 28;10:80-85.

*Mixed model longitudinal analysis of renal function parameters including the formula of Nankivell as well as the 4-variable MDRD formula with the fixed effects of treatment (SRL vs. CsA), time and the combination of the two confirmed a reduced decline of renal function in the SRL group during the long term follow up (supplementary Table 3). Adjustment for baseline value of the outcome variable was considered not appropriate because this adjustment for baseline differences is only advised when the expected values of the baseline value is the same for both groups which is the case in randomized trials but not in our observational setting in the SMART-DSA population.*

4. Survival analysis: The plots of graft and patient survival seems to violate the proportional Hazard assumption. The Schoenfeld Residuals Test should be done to test the proportional assumption. A more complex model should be used to incorporate the diferences in event-proportions during the follow-up like a time-dependent proportional Cox Model. The conclusion of better graft survival in the sirolimus group must be done by a Time Dependent Cox Model

Ref: Time-dependent covariates in the Cox proportional-hazards regression model. Fisher LD. Annu Rev Public Health. 1999;20:145-57.

*We performed several tests for the proportional hazard assumption:*

*Looking at the hazards of the therapy arms, the assumption of proportional hazards need not be rejected (Figure below).*

*Similarly, a plot log(-log(survival)) versus log survival time should show parallel lines if the assumption of proportional hazards is accepted. Even though, the graph for the CsA shows a certain deviation the assumption of proportional hazards again needs not be rejected (Figure below).*

*Another possibility to test for the assumption of proportional hazards is the introduction of a time dependent variable in the Cox model. We therefore included a variable “therapy arm*Log(time)” along with the “therapy arm”. Again the assumption of proportional hazards did not have to be rejected because the “therapy arm*Log(Time)” did not show a significant impact (p=0.5834).*

| Analysis of Maximum Likelihood Estimates | | | | | | | | | |
| --- | --- | --- | --- | --- | --- | --- | --- | --- | --- |
| Parameter |  | DF | Parameter Estimate | Standard Error | Chi-Square | Pr > ChiSq | Hazard Ratio | 95% Hazard Ratio Confidence Limits | |
| ARM | A:Srl | 1 | 0.58853 | 2.50501 | 0.0552 | 0.8143 | 1.801 | 0.013 | 244.266 |
| ARM*Log(T) |  | 1 | -0.33676 | 0.61405 | 0.3008 | 0.5834 | 0.714 | 0.214 | 2.379 |

| Linear Hypotheses Testing Results | | | | |
| --- | --- | --- | --- | --- |
|  | Label | Wald Chi-Square | DF | Pr > ChiSq |
|  | proportionality_test | 0.3008 | 1 | 0.5834 |

*An equivalent option to test for the assumption of proportional hazard is the graphical analysis of the Schoenfeld Residuals. In case this analysis fails to show a horizontal regression line a violation for the assumption of proportional hazards exists. When looking at the confidence interval, again the assumption of proportional hazards need not be rejected.*

| Supremum Test for Proportionals Hazards Assumption | | | | |
| --- | --- | --- | --- | --- |
| Variable | Maximum Absolute Value | Replications | Seed | Pr > MaxAbsVal |
| armi | 1.1049 | 1000 | 533916786 | 0.1040 |

***Summary***

*Even though certain deviations may exist, the analyses altogether imply that the assumption of proportional hazards can be accepted.*

*These tests were not included in the manuscript. We summarized these data in the “Results” section.*

*Analysis using time dependent covariates is not possible because of the anonymized data collection to the screening failures.*

5. Discussion. The authors should highlight the limitations of the very low rate of patients on the original immunosuppression at the end of 8-9 years follow-up. The conclusions about dnDSA must consider that the majority of the patients were in use of tacrolimus that was not the original immunosuppression.
The conclusion about better renal function in the sirolimus group should be re-analysed based on eGFR by MDRD or CKD-EPI. The conclusion about graft survival probably needs a Cox Model.

*The Discussion has been changed accordingly. As the SMART-Trial was designed in 2005/2006, the Nankivell Formula was used as primary parameter and it had been used in the two publications to the SMART-Trial before. Therefore, we feel the necessity to show Nankivell data here, as well. MDRD and CKD-EPI Data have also been shown in Tables 5 and 6.*

*Cox model has now been included for the reporting of the hazard ratio with confidence interval (supplementary Table 4).*

**Reviewer #2:** This is the long-term extension of a previous RCT aimed at evaluating early full conversion from a CNI- to an mTORi-based immunosuppressive protocol as compared with standard CNI-based immunosuppression in KTRs. Main findings of this study are that dnDSA developed similarly in both groups, and long-term renal graft function was better in SRL than in CNI, even though discontinuation rate was significantly higher under mTORi.
The manuscript is of interest for the audience of clinicians involved in the field of kidney transplantation, and findings refer to a very prolonged follow-up. Moreover it is well-written and statistical approach is robust.
However, it lacks originality, since main findings has been previously reported in larger cohorts. Moreover, full conversion from CNI to mTORi appears an old approach, since most studies aimed at addressing the protective role of mTORi on graft function are designed in order to compare mTORi and low exposure CNI vs standard exposure CNI therapy. Last, SRL vs CsA appears also a dated protocol comparison.

*The reviewer is right. This protocol is somewhat outdated. However, as we all know neither TAC nor combination therapy (mTOR-I + CNI) solve all the problems in renal transplantation. There are still situations for which patients can receive SRL or CsA.*

*We do not fully agree with the reviewer that our main findings have already been reported in larger cohorts. The importance of this trial is the long follow-up which to our knowledge none of the large randomized prospective trials have yet reached. Furthermore, we show that not only transplant function but also graft survival may benefit from mTOR-Is after such a long time.*

Other points:
As correctly acknowledged by the AA a veray graet incidence of drop-out is a limitation (about 50% of the original cohort), since can significantly affects reliability of results.
Is there any relationship between dnDSA development and eGFR changes over time? Indeed AA only present data on the relationship of endpoint eGFR and dnDNA.

*As we outlined in the manuscript and discussed as a limitation of this trial we measured dnDSA for this trial just once. Of course, there were some more measurements of dnDSA of these patients. However, these “other” measurements were usually dictated by clinical incidences and not systematically performed as required for a trial. Therefore, we do not have data to answer this question.*

Was AR, whose incidence was greater in the CsA group, associated with dnDSA development and/or graft survival?

*There is no significant correlation between DSA and the occurrence of acute rejections in this cohort. This information can also be found in Table 4 (Banff 4)*

Any data on proteinuria in the mTORi group?

*We included data on proteinuria on the SMART-DSA patients from the control visit in Table 7. There is data on proteinuria for the 12 and 36 months SMART Trial published in our manuscripts from 2010 and 2012. Here, the evidence indicated that proteinuria occurs more often early after transplantation under SRL vs. CsA. Following month 36 after Tx patients were no longer checked systematically for proteinuria. Therefore, we cannot make a valid statement on the development of proteinuria over time.*

**Reviewer #3**: This study reported long term results of the SMART trial (CNI free immunosuppression of SRL, MMF and Pred compared to CsA, MMF and Pred). The results also included development of denovo DSA in addition to GFR, graft and patient survival.
There is significant drop out which is reasonable for this long term observational study, however the reasons for drop out are not addressed or explained.

*Information on the dropouts can be found in Fig 1 and Table 1b.*

The therapy discontinuation in the Sirolimus group is significantly high compared to the controls (31% vs 66%), which would impact on the final conclusions. The number of patients with donor specific HLA Abs are also small to make a significant conclusion (5 vs 9), however as noted there is no impact type of immunosuppression on dnDSA.

The univariate analysis does not identify any significant risk factor as this study was a long term observation which by default had survived the worse outcomes irrespective of the risks and thus is a selection bias. This aspect or limitation should be explained in the discussion.

The benefits of sirolimus in preserving GFR and long term graft survival is demonstrated again in this follow up.

I would recommend addressing these limitations in the discussion and also revising the the language in the Introduction and Discussion sections to conventional manuscript format.

*The discussion has been modified accordingly*

**Reviewer #4**: This study describes the long-term results (about 9 years) of SMART-Trial, focusing on the development of dnDSA.

Despite the widespread importance of knowing long-term efficacy and safety results of immunosuppressive strategies with mTOR inhibitors, some aspects should be pointed out:

1. The strategy used in SMART-Trial was promising in 2006, but it is currently an exception immunosuppressive regimen: CsA instead of TAC + preemptive conversion rather than mTORi de novo + loading-dose SRL. This makes these results less interesting today.

*As outlined before, the SMART Trial was designed in 2005/2006. According to the OPTN report > 90 % of patients are put on TAC/MMF +/- ST. This regimen has been predominantly used for the past 10-15 yrs. In recent years mTOR-I+CNI combination therapy has increased somewhat. Nonetheless, both the SRL as well as the CsA can still have an indication. For these, our data may be useful all the more considering the long follow-up and conclusion.*

2. Focusing on dnDSA as the primary objective, trying to correlate the results with the immunosuppressive regimen, does not seem appropriate, since:
a) Only 34 of 140 patients originally enrolled were on therapy.
b) Over such a long follow-up period, many non-evaluated variables may interfere on results, such as: drug conversions, immunosuppression exposure (doses/concentration / variability), and compliance with treatment.
c) There is no information on pre-formed DSA, which makes it difficult to conclude that a first measured DSA is de novo.

I believe a more complete descriptive analysis of efficacy and safety aspects of the 140 patients originally enrolled in SMART trial would be most useful.

*We agree that the* *primary objective can be questioned. On the other hand, DSA is a still unsolved issue and different results have been published in the context of mTOR-I- and CNI-use. We believe it is of interest for the transplant community and therefore justified to show long term data in this regard.*

*As the reviewer correctly states only a minority of the originally included patients were still “on therapy”. In addition, other variables may have interfered in the meantime because a very long time has passed since the transplant. The strength of this trial is the unusual long follow-up with “real-life” data on once randomized and prospectively followed patients.*

*All patients had a negative T- and B-cell-complement dependent cytotoxicity crossmatch before transplantation. None of the patients with post-transplant de novo DSA had pre-transplant DSA.*

Minor issues:
1. I suggest inserting the legends of abbreviations used in the tables and figures

2. I suggest pre-define the abbreviations used throughout the text: Tx, n.s., pts, Abs, SCr-Tk+7, CAN, DCGS, etc

*This has been amended*

3. I suggest caution in concluding on renal function based on formulas not used today, as Cockroft-Gault and Nankivell.

*MDRD and now also CKD-Epi have been included.*

4. I suggest adjusting the analysis of renal function for deaths, graft losses and follow-up losses. (mixed models longitudinal analyses)

*This would be the way for a common regular prospective 12 months’ study. However, we don’t believe this to be justified for this trial where there are many more deaths, graft losses etc. due to the long follow-up.*

5. I suggest to pre-define the adverse events listed in Table 7.

*We are not sure what is meant by this. Could you please specify?*

6. I suggest that the Discussion session should not be used to repeat results but for critical analysis of study findings.

*The discussion has been modified.*

7. I suggest improving Figure 1 to better demonstrate how many and when patients died, lost their grafts, lost the follow-up and discontinued drugs.

*We pondered extensively over this. As a matter of fact, Figure 1 contains a lot of information: it shows the original trial plan, the follow-up trial (36 months), the current trial plan and the dropouts of the patients and the reasons thereof. Unfortunately, we could not think of another way to show all these data in a more comprehensible way.*

**Reviewer #5**: The authors updated a German RCT (SMART Trial) with about half (71/140) of the original study group. Blood samples were obtained an average of 8.7 years after initial enrollment in order to screen for the development of DSA. They concluded that “An early conversion to SRL does not result in an increased incidence of dnDSA nor increased long-term risk for the recipient. Transplant function remains improved with benefits for the graft survival.” The important signals reported for the studied survivors were no significant increase in DSA (SRL 5/38, 13.2% vs. CsA 9/33, 27.3%; P=0.097) with an improved eGFR (SRL 64.37 vs. CsA 53.19 ml/min/1.73m2, p=0.044), for those converted to sirolimus. Additional signals that are very encouraging were trends to lower rates of de novo cancers and perhaps fewer viral infections.

In many prior trials using mTORi based immunosuppression to reduce or eliminate CNI drugs very good outcomes were reported for those that tolerated the mTORi. However, prior trials were often plagued by dropouts and investigator switches off the mTORi drugs for various side effects including oral lesions, dyslipidemia, edema, lymphoceles, wound disruptions/hernias, GI intolerance, etc. Many such switches seemed arbitrary and inconsistent, and in effect prevented the formation of firm conclusions from many well-designed trials.

1. While offering some reassuring long-term data that DSA formation is not excessive for those recipients remaining on an mTORi in a CNI free regimen, this sub study only reports 31% of the SRL recipients still taking the drug, and many were on another CNI. So again, why were so many switched? A small table listing the reasons would be informative. Just listing late adverse events is not as informative as listing those that caused a change in drug delivery.

2. No doubt many of the switches off SRL were done due to the side effects caused similarly by mycophenolate mofetil; especially bone marrow suppression and GI intolerance. This should also be noted.

*1 and 2: We have now included more information on these patients that were switched (See also supplementary Tables 1 and 2).*

3. Any critical analysis of long-term renal allograft function, especially on an mTORi requires demonstration of the presence and magnitude of proteinuria. I found no such data which is a major limitation of this paper. This should be provided, at least UA dipsticks, or featured as a major limitation.

*See also comment to the reviewer above…..*

*We included data on proteinuria on the SMART-DSA patients from the control visit (see Table 7 adverse events). There is data on proteinuria for the 12 and 36 months SMART Trial published in our manuscripts from 2010 and 2012. Here, the evidence indicated that proteinuria occurs more often at these earlier timepoints after transplantation under SRL vs. CsA. Following month 36 up to the last visit patients were not checked for proteinuria systematically, Therefore, we cannot really comment on the development of proteinuria over time.*

4. In addition to #3, the absence of histology of the grafts also limits the ability to make firm conclusions on the impact of CNI free status on graft stability, This limitation should be provided as well.

*The discussion has been amended accordingly.*

**Reviewer #6**: This paper looks at a long term follow-up of a clinical trial. Roughly half the patients originally enrolled are available for this follow-up study - the most common reasons for not being in the long-term follow-up study are death and progression.

It is reassuring to note there appears to be no differential dropout by arm; but it would also be useful in Table 1a to look at the comparison of the ITT group and the long-term group, because this will show how the trial population has changed over time.

*We now included the data to the original ITT patients*

Please do not use p=n.s. but give actual p-values in the text as they are there in the Tables. The precision should be the same also - 1sf below p=0.01 is fine.

*Changes have been made accordingly*

In Table 5-6 there are clear differences between arms in the "on therapy" population - I think these figures should be given in summary only and not tested given the known imbalance (p=0.004) identified earlier - this would invalidate the underlying statistical assumption.

*We are aware of this problem. However, for descriptive reasons we decided to leave the p-values for the “on-therapy” group in the Table.*

Please give HR & CI for survival to see if this is evidence of lack of effect or lack of evidence of effect. Similarly for graft survival - if p<.05 represents significance then please do not talk of trends to wards or differences when p>.05.

*HR & CI were included in the results section and the Figure Legends*

Figure 3 and 4 require total numbers of events per arm to show the power to detect a difference.

*Total numbers have been included in the Figure Legends*
