# Supplementary material for: Early conversion to a CNI-free immunosuppression with SRL after renal transplantation—Long-term follow-up of a multicenter trial
Source: PLoS One. 2020 Aug 5;15(8):e0234396. doi: 10.1371/journal.pone.0234396 (PMC7406080; doi:10.1371/journal.pone.0234396)
Supplement: Attachment — Submitted filename: 2020_Plos_One.docx [file pone.0234396.s017.docx]

Authors have clarified several points raised by reviewers. They also included important information in the text.

1. **However, some answers to the reviewers' questions should also be highlighted in the text so that the reader can also access. Example:**

*Rewier comment:*

*“Focusing on dnDSA as the primary objective, trying to correlate the results with the immunosuppressive regimen, does not seem appropriate, since:*

*a) Only 34 of 140 patients originally enrolled were on therapy.*

*b) Over such a long follow-up period, many non-evaluated variables may interfere on results, such as: drug conversions, immunosuppression exposure (doses/concentration / variability), and compliance with treatment.*

*c) There is no information on pre-formed DSA, which makes it difficult to conclude that a first measured DSA is de novo.*

*I believe a more complete descriptive analysis of efficacy and safety aspects of the 140 patients originally enrolled in SMART trial would be most useful.”*

*Authors response:*

*We agree that the primary objective can be questioned. On the other hand, DSA is a still unsolved issue and different results have been published in the context of mTOR-I- and CNI-use. We believe it is of interest for the transplant community and therefore justified to show long term data in this regard.*

*As the reviewer correctly states only a minority of the originally included patients were still “on therapy”. In addition, other variables may have interfered in the meantime because a very long time has passed since the transplant. The strength of this trial is the unusual long follow-up with “real-life” data on once randomized and prospectively followed patients.*

*All patients had a negative T- and B-cell-complement dependent cytotoxicity crossmatch before transplantation. None of the patients with post-transplant de novo DSA had pre- transplant DSA.*

2. **Abstract – Conclusion:** “An early conversion to SRL does not result in an increased incidence of dnDSA nor increased long-term risk for the recipient. Transplant function remains improved with benefits for the graft survival”

Given the uncontrolled nature of this study (with a lot of limitation precluding definitive conclusion), best to say that: an early conversion to SRL did not result…

Your results also did not show that graft function remains improved…

3. **Pag 16, line 295. “Beginning at 8-9 years after the transplantation, Kaplan-Meier curves show a particularly increased death censored failure rate for the CsA treated patients (p=0.064, Figure 4).**

I don't think it is possible to say this, because:

1. the number of patients at risk is very small after 100 months

2. p> 0.05

3. I'm not sure if Log-rank is the ideal test to compare this curves

4. **Your conclusion on p>0.05 are not uniform.**

Sometimes p>0.05 are significant… sometimes it is not… I suggest maintaining consistency in this interpretation.

Examples:

Suppl Table 1: “Exposure time to Tacrolimus was not significantly different between the groups. (p=0.09)”

Pág. 15, l.269: “GFR comparison of month 3 after Tx to most recently (104+9 months) revealed a more pronounced deterioration in the CsA group (MDRD: -0.87 ± 14.58 ml/min/1.73m2 SRL vs. -8.26 ± 18.04 ml/min/1.73 m2 CsA; p=0.07; CKD-EPI: -2.08 ± 15.39 ml/min/1.73 m2 SRL vs. -9.91 ± 18.59 ml/min/1.73 m2 CsA; p=0.06; Table 6).”

Pag 16, line 295: “Beginning at 8-9 years after the transplantation, Kaplan-Meier curves show a particularly increased death censored failure rate for the CsA treated patients (p=0.064, Figure 4)”

5. **I suggest to edit your Suppl Table 2 to make the comparison between groups more visible**

| Category |  | A: SRL | B: CsA |
| --- | --- | --- | --- |
| Rejection |  | X | X |
| Deteriorating Tx function |  | X | X |
| Rising Creatinine |  | X | X |
| Fibrosis, progressive degeneration |  | X | X |
| Proteinuria |  | X | X |
| etc |  | X | X |
| Miscellaneous |  | X | X |
| Diarrhea  Hyperlipidemia |  | X | X |
| HLA-antibodies  Diarrhea  etc |  | X  X | X  X |

6. **Suppl Table 4:** “According to this analysis the risk to lose the graft (death censored graft survival; DCGS) is smaller under SRL by a factor of 0.461 (95%CI: 0.199 – 1.069)”.

This affirmation is not in accordance with the demonstrated 95%CI

7. Please define the following adverse events:

- Infections: Any infection? Viral infection? Infection requiring hospitalization?

- Metabolic disorders: diabetes? Dyslipidemia? Hyperuricemia? All disorders?

8. **What does “SCr-Tk+7” mean?**
9. **Pag 18, line 307: p=n. s.** Please include the p value.
