# Supplementary material for: Early conversion to a CNI-free immunosuppression with SRL after renal transplantation—Long-term follow-up of a multicenter trial
Source: PLoS One. 2020 Aug 5;15(8):e0234396. doi: 10.1371/journal.pone.0234396 (PMC7406080; doi:10.1371/journal.pone.0234396)
Supplement: Attachment — Submitted filename: Andrassy SMART Response II.docx [file pone.0234396.s018.docx]

| Klinikum der Universität München ∙ Klinik für Allgemein-, Viszeral-, Transplantations-chirurgie ∙ CAMPUS GROSSHADERN ∙ Marchioninistr. 15 ∙ D-81377 München | |  | **Prof. Dr. med. J. Andrassy**  [Joachim.andrassy@med.uni-muenchen.de](mailto:Joachim.andrassy@med.uni-muenchen.de)  Sekretariat  Tel.: +49 89 4400-76561  Fax: +49 89 4400-78894  <http://gch.klinikum.uni-muenchen.de>  Postanschrift:  Klinikum der Universität München  Klinik für Allgemein-, Viszeral-, Transplantationschirurgie  Marchioninistr. 15  D-81377 München |
| --- | --- | --- | --- |
| Prof. Justyna Gołębiewska  Academic Editor  Plos ONE | |  |  |
| Ihr Zeichen: | Unser Zeichen: |  | München, den 04.05.20 |

PONE-D-19-25265
Early conversion to a CNI-free immunosuppression with SRL after renal Transplantation – long-term follow-up of a multicenter trial
PLOS ONE

6. Review Comments to the Author

Please use the space provided to explain your answers to the questions above. You may also include additional comments for the author, including concerns about dual publication, research ethics, or publication ethics. (Please upload your review as an attachment if it exceeds 20,000 characters)

Reviewer #1: I considered that the authors address all the suggestions. The statistic corrections were made. Also, the limitations were discussed in more detail. I considered that the manuscript may be considered for publication in the current revised form.

Reviewer #2: The AA adequately addressed all the concerns raised by this reviewer.

The manuscript was significantly improved

Reviewer #3: The authors have addressed the criticisms about the dropouts in their revision and also explained in the reason for lack of data on proteinuria after 36 months. The manuscript has also been changed to meet the conventional format. The authors have also changed the estimation of GFR to CKD-epi and MDRD. This study has merit in terms of the long follow up, and the limitation would be as mentioned buy the authors the analysis of DSAs was limited to 49% of the study population.

Reviewer #4: Authors have clarified several points raised by reviewers.

They also included important information in the text.

Abstract – Conclusion: “An early conversion to SRL does not result in an increased incidence of dnDSA nor increased long-term risk for the recipient. Transplant function remains improved with benefits for the graft survival”

Given the uncontrolled nature of this study (with a lot of limitation precluding definitive conclusion), best to say that: an early conversion to SRL did not result…

*This has been changed*

Your results also did not show that graft function remains improved…

*We disagree. Figure 2 and Table 5 both show significant differences. However, it is also clear that all statistical analyses to the “secondary” objectives have to be regarded as descriptive. Only the analyses to the primary objective (dnDSA) is confirmatory. This is described in further detail in response to your next query.*

Pag 16, line 295. “Beginning at 8-9 years after the transplantation, Kaplan-Meier curves show a particularly increased death censored failure rate for the CsA treated patients (p=0.064, Figure 4).

I don't think it is possible to say this, because:

1. the number of patients at risk is very small after 100 months

2. p> 0.05

3. I'm not sure if Log-rank is the ideal test to compare this curves

*As described in the methods section this is a descriptive analysis and therefore p-values must not be used in a confirmatory sense. For descriptive purposes any p-value can be used and as the logrank test is usually reported we followed this pattern in planning the analysis. As with the log-rank test differences at all time intervals are weighted equally, this is a reasonable choice for most situations.*

*You may consider the Wilcoxon test where differences are weighted by the number at risk at the time, thus giving more weight to differences that occur earlier in follow-up time. But in our opinion this is not the best solution for a situation, where events in the early follow-up are generally rare and differences could be expected in the long run only. Other nonparametric tests using other weighting schemes are available and we present the results below:*

| Test of Equality over Strata | | | |
| --- | --- | --- | --- |
| Test | Chi-Square | DF | Pr > Chi-Square |
| Log-Rank | 3.4197 | 1 | 0.0644 |
| Wilcoxon | 1.5991 | 1 | 0.2060 |
| Tarone | 2.2807 | 1 | 0.1310 |
| Peto | 2.9863 | 1 | 0.0840 |
| Modified Peto | 2.9335 | 1 | 0.0868 |
| Fleming(1) | 3.0481 | 1 | 0.0808 |

*Finally, we should overcome the temptation to chose the test that best suits the intended interpretation. We still would report the logrank result, as this test is familiar to most readers. In our opinion it should be sufficient to point out the limited relevance of a p-value and let the curves speak for their own.*

Suppl Table 4: “According to this analysis the risk to lose the graft (death censored graft survival; DCGS) is smaller under SRL by a factor of 0.461 (95%CI: 0.199 – 1.069)”.

This affirmation is not in accordance with the demonstrated 95%CI

*We changed the wording to “According to this analysis the hazard ratio for graft loss was 0.461 (95%CI: 0.199-1.069) in patients once randomized to SRL compared to the CsA group.”*

Please define the following adverse events:

- Infections: Any infection? Viral infection? Infection requiring hospitalization?

- Metabolic disorders: diabetes? Dyslipidemia? Hyperuricemia? All disorders?

*As can be seen from the table below the detailed workup does not add significance to the general data due to the low numbers. Therefore, we feel the inclusion of a more detailed description of the infections and metabolic disorders not justified.*

|  | | **A:Srl** | | **B:CsA** | | **Z:ALL** | |
| --- | --- | --- | --- | --- | --- | --- | --- |
| **socName** | **ptName** | **N** | **%** | **N** | **%** | **N** | **%** |
| Any SOC |  | 28 (74%) | 22 (67%) | 50 (70%) | 28 (74%) | 22 (67%) | 50 (70%) |
| ... |  |  |  |  |  |  |  |
| Infections and infestations | ALL | 9 | 23.68 | 7 | 21.21 | 16 | 22.54 |
|  | Bronchopneumonia | 1 | 2.63 | . | . | 1 | 2.63 |
|  | Campylobacter gastroenteritis | . | . | 1 | 3.03 | 1 | 3.03 |
|  | Cellulitis | 1 | 2.63 | . | . | 1 | 2.63 |
|  | Diverticulitis | 1 | 2.63 | . | . | 1 | 2.63 |
|  | Enterocolitis bacterial | . | . | 1 | 3.03 | 1 | 3.03 |
|  | Epstein-Barr virus infection | 1 | 2.63 | . | . | 1 | 2.63 |
|  | Erysipelas | 1 | 2.63 | . | . | 1 | 2.63 |
|  | Gastroenteritis | 2 | 5.26 | 3 | 9.09 | 5 | 7.04 |
|  | Herpes zoster | 2 | 5.26 | 1 | 3.03 | 3 | 4.23 |
|  | Lobar pneumonia | 2 | 5.26 | . | . | 2 | 5.26 |
|  | Mastoiditis | . | . | 1 | 3.03 | 1 | 3.03 |
|  | Pneumonia | 2 | 5.26 | . | . | 2 | 5.26 |
|  | Urinary tract infection | . | . | 2 | 6.06 | 2 | 6.06 |
|  | Urosepsis | 1 | 2.63 | 1 | 3.03 | 2 | 2.82 |
| ... |  |  |  |  |  |  |  |
| Metabolism and nutrition disorders | ALL | 5 | 13.16 | 4 | 12.12 | 9 | 12.68 |
|  | Abnormal loss of weight | . | . | 1 | 3.03 | 1 | 3.03 |
|  | Diabetes mellitus | 1 | 2.63 | 1 | 3.03 | 2 | 2.82 |
|  | Dyslipidaemia | 1 | 2.63 | . | . | 1 | 2.63 |
|  | Gout | 1 | 2.63 | . | . | 1 | 2.63 |
|  | Hypercalcaemia | . | . | 1 | 3.03 | 1 | 3.03 |
|  | Hypercholesterolaemia | 2 | 5.26 | 1 | 3.03 | 3 | 4.23 |
|  | Hyperuricaemia | 1 | 2.63 | . | . | 1 | 2.63 |
|  | Obesity | 1 | 2.63 | . | . | 1 | 2.63 |
| ... |  |  |  |  |  |  |  |
| Cardiac/vascular disorders | ALL | 8 | 21.05 | 3 | 9.09 | 11 | 15.49 |
|  | Angina pectoris | 1 | 2.63 | . | . | 1 | 2.63 |
|  | Aortic stenosis | . | . | 1 | 3.03 | 1 | 3.03 |
|  | Aortic valve incompetence | 1 | 2.63 | . | . | 1 | 2.63 |
|  | Aortic valve stenosis | . | . | 1 | 3.03 | 1 | 3.03 |
|  | Atrial fibrillation | 2 | 5.26 | 1 | 3.03 | 3 | 4.23 |
|  | Atrial flutter | 1 | 2.63 | 1 | 3.03 | 2 | 2.82 |
|  | Cardiac failure | 1 | 2.63 | . | . | 1 | 2.63 |
|  | Intermittent claudication | . | . | 1 | 3.03 | 1 | 3.03 |
|  | Myocardial infarction | 1 | 2.63 | . | . | 1 | 2.63 |
|  | Peripheral arterial occlusive disease | 1 | 2.63 | . | . | 1 | 2.63 |
|  | Peripheral artery stenosis | 1 | 2.63 | . | . | 1 | 2.63 |
| Patients | . | 38 | 100.00 | 33 | 100.00 | 71 | 100.00 |

What does “SCr-Tk+7” mean?

***S****erum* ***cr****eatinine* ***7*** *days after the* ***t****imepoint of* ***c****onversion*

*We attached an asterisk with an explanation underneath the table.*

Pag 18, line 307: p=n. s. Please include the p value.

*This has been changed*

Reviewer #5: The authors have provided extension data from a prior RCT termed SMART including 71 of the original ITT 140 kidney transplant recipients with a mean 8.7 year follow-up. The aim of this extension was to detect de novo DSA formation comparing those on a CNI free regimen after a 3-week switch from CsA to Sirolimus to those that remained on CsA; for each MMF/Cs continued. They report that a statistically significant increase in dnDSA was not detected (SRL 5/38, 13.2% vs. CsA 9/33, 27.3%; P=0.097) and GFR remained improved under SRL with (64 vs. 53 ml/min/1.73m2, p=0.044). Although graft failure and skin tumors were less for those on SRL, the difference was not statistically significant. From these data they concluded “An early conversion to SRL does not result in an increased incidence of dnDSA nor increased long-term risk for the recipient. Transplant function remains improved with benefits for the graft survival.”

The study and its results, like many other long-term kidney transplant trials, especially including mTORi suffer from incomplete and perhaps unreliable patient selection and retention. To their credit the authors freely point out that the use of mTORi have been plagued by a lack of tolerability for many patients, and the very inconsistent role more peripheral investigators play in drug switching and dose changes. Their rather sober tally of only “31.6% (12/38) once randomized to receive SRL compared to 66.7% (22/33) started on CsA were still on the original immunosuppressant. The majority had stopped the SRL relatively early as only 40.6% had remained “on therapy” after the first 3 years.” That said mean 8.7 year follow-up data is about the best one could expect to see and MMF switches and changes can be 50% or more as well. More generalized themes are more likely to be true.

Comments

1. The paper often uses 3-month for the time of conversion to SRL—wasn’t it 3 weeks? Fix this in the narrative.

*The reviewer is right that the conversion to SRL took place within 3 weeks after the transplantation. However, the first time point our “long-term” data were compared with is 3 months after transplantation. We screened the manuscript but no corrections were necessary in this regard.*

2. Fix your Figure 1 flow sheet and list how many were on sirolimus-MMF-Cs and how many were on CNI-MMF-Cs. How many were truly CNI-free?

*This has been changed accordingly*

3. Those who remain CNI-free on an mTORI usually demonstrate a statistically significant reductions in both skin and other cancers over time, especially after 3 years. The lack of significance in your study, I speculate, is due to the very few that are CNI-free and the larger numbers that switched back to a CNI. Comment.

*As pointed out in Table 7 (adverse events) and down below only a few patients developed these AEs after 36 months after the transplantation. We analyzed the results for the “on therapy” group as suggested. We inserted the results for malignancy in the text of the results section.*

**Incidence of Malignancies**

|  | A: SRL | B: CsA | P-value |
| --- | --- | --- | --- |
| **SMART-DSA** | **N=38** | **N=33** |  |
| Malignancies | 1 (2.63) | 5 (15.15) | 0.0900 |
| **On Therapy** | **N=12** | **N=22** |  |
| Malignancies | 0 (0..00) | 5 (27.27) | 0.0691 |

**Rejections**

|  | A: SRL | B: CsA | P-value |
| --- | --- | --- | --- |
| **SMART-DSA** | **N=38** | **N=33** |  |
| Rejection | 0 | 3 | 0.0955 |
| BPAR | 0 | 3 | 0.0955 |
| Banff 4 | 0 | 0 | - |
| **On Therapy** | **N=12** | **N=22** |  |
| Rejection | 0 | 0 | - |
| BPAR | 0 | 0 | - |
| Banff 4 | 0 | 0 | - |

**Incidence of Proteinuria**

|  | A: SRL | B: CsA | P-value |
| --- | --- | --- | --- |
| **SMART-DSA** | **N=38** | **N=33** |  |
| Proteinuria during the study ^*)^ | 8 (21.05) | 8 (24.24) | 0.7825 |
| Proteinuria at the endo f study visit ^**)^ | 3 (7.89) | 7 (21.21) | 0.1712 |
| **On Therapy** | **N=12** | **N=22** |  |
| Proteinuria during the study ^*)^ | 1 (8.33) | 4 (18.18) | 0.6347 |
| Proteinuria at the endo f study visit ^**)^ | 1 (8.33) | 3 (13.64) | 1.0000 |

^*)^ reported as AE, ^**)^ by clinically significant laboratory value

4. The Discussion should add a comment that dnDSA may have more to do with the stringency of HLA mismatches rather than the choice of maintenance immuno-suppression. The emerging powerful message from eplet matching would seem to support this hypothesis. AJT 2013; 13: 3114–3122 and JASN 2017; 28: 3353–3362.

*Development of dnDSA certainly has to do with immunogenicity for which eplets, of course tell a more complete story. And personally, I do agree with the reviewer that this probably has a more pronounced effect than the immunosuppressive drug used. However, as of yet this has to be shown. Even in the above described cited paper (JASN 2017) not only the eplet-mismatch but also the immunosuppressive drug (CsA vs. TAC) and the trough levels thereof significantly correlated with the incidence of dnDSA.*

*We included a paragraph to the eplet-matching in the discussion.*

5. The authors really need to cull out the 40% or so that began and remained on SRL-MMF-Cs for the 8 years. What were their eGFR and urine protein excretion? How many experienced biopsy confirmed rejection and did any of these develop cancer?

*eGFR-data for “on therapy” patients is in Tables 5&6. As described above, we did analyze the “on therapy” data for the adverse events and included data to malignancy. However, no further significant details could be found regarding rejections and proteinuria, most likely due to the low numbers.*

Reviewer #6: (No Response)
